# Retrospective Analysis of the Treatment Outcome in Myeloid Leukemia of Down Syndrome in Polish Pediatric Leukemia and Lymphoma Study Group From 2005 to 2019

**DOI:** 10.3389/fped.2020.00277

**Published:** 2020-06-19

**Authors:** Malgorzata Czogala, Katarzyna Pawinska-Wasikowska, Teofila Ksiazek, Barbara Sikorska-Fic, Michal Matysiak, Jolanta Skalska-Sadowska, Jacek Wachowiak, Anna Rodziewicz-Konarska, Alicja Chybicka, Katarzyna Myszynska-Roslan, Maryna Krawczuk-Rybak, Dominik Grabowski, Jerzy Kowalczyk, Lucyna Maciejka-Kemblowska, Elzbieta Adamkiewicz-Drozynska, Katarzyna Bobeff, Wojciech Mlynarski, Renata Tomaszewska, Tomasz Szczepanski, Joanna Pohorecka, Agnieszka Chodala-Grzywacz, Grazyna Karolczyk, Agnieszka Mizia-Malarz, Katarzyna Mycko, Wanda Badowska, Karolina Zielezinska, Tomasz Urasinski, Magdalena Nykiel, Mariola Woszczyk, Malgorzata Ciebiera, Radosław Chaber, Szymon Skoczen, Walentyna Balwierz

**Affiliations:** ^1^Department of Pediatric Oncology and Hematology, Institute of Pediatrics, Jagiellonian University Medical College, Kraków, Poland; ^2^Department of Pediatric Oncology and Hematology, University Children Hospital, Kraków, Poland; ^3^Department of Medical Genetics, Institute of Pediatrics, Jagiellonian University Medical College, Kraków, Poland; ^4^Department of Pediatrics, Hematology and Oncology, Medical University of Warsaw, Warsaw, Poland; ^5^Department of Pediatric Oncology, Hematology and Transplantology, Poznan University of Medical Sciences, Poznań, Poland; ^6^Department of Bone Marrow Transplantation, Pediatric Oncology and Hematology, Medical University of Wroclaw, Wrocław, Poland; ^7^Department of Pediatric Oncology and Hematology, Medical University of Bialystok, Bialystok, Poland; ^8^Department of Pediatric Hematology, Oncology and Transplantology, Medical University of Lublin, Lublin, Poland; ^9^Department of Pediatrics, Hematology and Oncology, University Medical Centre, Gdańsk, Poland; ^10^Department of Pediatrics, Oncology, Hematology and Diabetology, Medical University of Lodz, Łódź, Poland; ^11^Department of Pediatrics Hematology and Oncology, Medical University of Silesia, Zabrze, Poland; ^12^Paediatric Department of Hematology and Oncology, Regional Polyclinic Hospital in Kielce, Kielce, Poland; ^13^Department of Oncology, Hematology and Chemotherapy, John Paul II Upper Silesian Child Heath Centre, The Independent Public Clinical Hospital No. 6 of the Medical University of Silesia in Katowice, Katowice, Poland; ^14^Department of Pediatrics and Hematology and Oncology, Province Children's Hospital, Olsztyn, Poland; ^15^Department of Pediatrics, Hematology and Oncology, Pomeranian Medical University, Szczecin, Poland; ^16^Department of Pediatrics, Hematology and Oncology, City Hospital, Chorzów, Poland; ^17^Department of Pediatric Oncohematology, Clinical Province Hospital of Rzeszów, Rzeszow, Poland

**Keywords:** myeloid leukemia, down syndrome, children, treatment results, genetic characteristics

## Abstract

**Background:** Children with Down syndrome (DS) have increased risk of myeloid leukemia (ML), but specific treatment protocols ensure excellent outcome. This study was a retrospective analysis of the treatment results and genetic characteristics of ML of DS (ML-DS) in Poland from 2005 to 2019.

**Methods:** All 54 patients with ML-DS registered in the Polish Pediatric Leukemia and Lymphoma Study Group in analyzed period were enrolled to the study. There were 34 children treated with Acute Myeloid Leukemia–Berlin-Frankfurt-Munster 2004 Interim Protocol (group I) and 20 patients treated with ML-DS 2006 Protocol (group II). In the first protocol, there was reduction of the antracyclines doses and intrathecal treatment for ML-DS compared to non-DS patients. In the second protocol, further reduction of the treatment was introduced (omission of etoposide in the last cycle, no maintenance therapy).

**Results:** Probabilities of 5-year overall survival (OS), event-free survival (EFS), and relapse-free survival in the whole analyzed group were 0.85 ± 0.05, 0.83 ± 0.05, and 0.97 ± 0.03, respectively. No significant differences were found between two protocols in the terms of OS and EFS (0.79 ± 0.07 vs. 0.95 ± 0.05, *p* = 0.14, and 0.76 ± 0.07 vs. 0.95 ± 0.05, *p* = 0.12, respectively). All deaths were caused by the treatment-related toxicities. Reduction of the treatment-related mortality was noticed (20% in group I and 5% in group II). The only one relapse in the whole cohort occurred in the patient from group I, older than 4 years, without *GATA1* gene mutation. He was treated successfully with IdaFLA cycle followed by hematopoietic stem cell transplantation from matched sibling donor. No significant prognostic factor was found in the study group probably due to low number of patients in the subgroups.

**Conclusions:** The study confirms that the reduced intensity protocols are very effective in ML-DS patients. The only cause of deaths was toxicities; however, systematic decrease of the treatment-related mortality was noticed.

## Introduction

Children with Down syndrome (DS) have increased risk of myeloid leukemia (ML) compared to children without DS (150-fold before the age of 5 years) (1). Myeloid leukemia in DS children (ML-DS) is characterized by several unique features. Approximately 50% of patients are diagnosed within the first year of life, and only 1–2% at 4 years or older (1). It shows a high prevalence of the acute megakaryocytic leukemia phenotype (AMKL), which is rare in non-DS acute myeloid leukemia (AML) (2, 3). Myeloid leukemia of DS can be preceded by transient abnormal myelopoiesis (TAM) observed in approximately 10% of neonates with DS (4, 5). Approximately 20 to 30% of TAM can progress to ML-DS (5, 6). Both TAM and ML-DS, especially AMKL, are associated with mutations of the hematopoietic transcription factor GATA1 (7–9). Development of ML-DS is frequently preceded by myelodysplastic phase characterized by thrombocytopenia and anemia. Myeloid neoplasms of DS are not subclassified into myelodysplastic syndrome (MDS) or AML because they have a similar behavior independently of blast cell count (10). In case of evidence for leukemic blasts in the bone marrow, it is recommended that children with DS should be diagnosed with AML, even if the blast threshold of 20% is not reached (11). According to the World Health Organization 2008 and 2016 classifications, both MDS and AML in children with DS are classified as a separate entity—ML of DS (ML-DS) (10, 12).

Myeloid blasts in children with DS have high drug sensitivity especially to cytarabine and anthracyclines (13). That determines excellent response to the treatment. The risk of the therapy-associated toxicities is higher in children with DS compared to other patients with AML (14). Introduction of the DS-specific therapeutic protocols with reduced intensity of the treatment resulted in excellent outcome (14–17).

The aim of the study was retrospective analysis of the treatment results and genetic characteristics of pediatric ML-DS patients treated in Poland from 2005 to 2019.

## Patients and Methods

**Figure 1:**
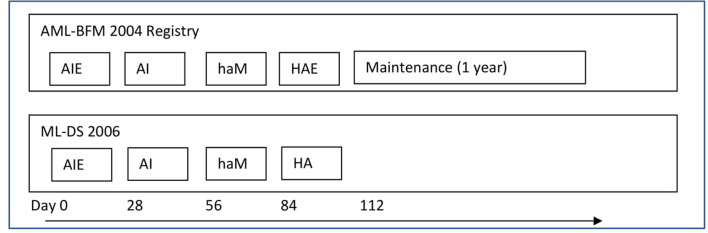
The treatment protocols. AML-BFM 2004 Registry—group I (reduced intensity arm for children with ML-DS)—group I: AIE (cytarabine 100 mg/m^2^/day [days 1–2] and 100 mg/m^2^ every 12 h [days 3–8], idarubicin 8 mg/m^2^/day [days 3, 5, and 7], and etoposide 150 mg/m^2^/day [days 6–8]); AI (cytarabine 500 mg/m^2^/day [days 1–4] and idarubicin 5 mg/m^2^/day [days 3 and 5]); haM (cytarabine 1 g/m^2^ every 12 h [days 1–3] and mitoxantrone 7 mg/m^2^/day [days 3–4]); HAE (cytarabine 3 g/m^2^/every 12 h [days 1–3], etoposide 125 mg/m^2^/day [days 2–5]). Maintenance therapy lasting 1 year: thioguanine 40 mg/m^2^/day orally, cytarabine 40 mg/m^2^/day intravenously for 4 consecutive days, every 4 weeks. Intrathecal cytarabine (CNS prophylaxis)—in the each intensive treatment block, not during maintenance therapy (in total, six aged adapted doses−20 to 40 mg per dose). The cumulative doses: 29,400 mg/m^2^ cytarabine, 950 mg/m^2^ etoposide, 34 mg/m^2^ idarubicin, and 14 mg/m^2^ mitoxantrone. ML-DS 2006—group II: AIE (cytarabine 100 mg/m^2^/day [days 1–2] and 100 mg/m^2^ every 12 h [days 3–8], idarubicin 8 mg/m^2^/day [days 3, 5, and 7], and etoposide 150 mg/m^2^/day [days 6–8]); AI (cytarabine 500 mg/m^2^/day [days 1–4] and idarubicin 5 mg/m^2^/day [days 3 and 5]); haM (cytarabine 1 g/m^2^ every 12 h [days 1–3] and mitoxantrone 7 mg/m^2^/day [days 3–4]); HA (cytarabine 3 g/m^2^/every 12 h [days 1–3]). Intrathecal cytarabine (CNS prophylaxis)—at the start of each treatment block (in total, four aged adapted doses−20–40 mg per dose). The cumulative doses: 27,400 mg/m^2^ cytarabine, 450 mg/m^2^ etoposide, 34 mg/m^2^ idarubicin, and 14 mg/m^2^ mitoxantrone.

From January 2005 to December 2019, there were 54 newly diagnosed ML-DS patients registered in the Polish Pediatric Leukemia and Lymphoma Study Group (PPLLSG) registry. They were treated in 16 pediatric oncology centers in Poland. All were eligible to the study. Inclusion criteria included age up to 18 years, diagnosis of DS and ML, and written informed consent. Exclusion criteria comprised accompanying diseases that do not allow ML-DS therapy. Thirty-four children were treated with DS-specific arm of the Acute Myeloid Leukemia–Berlin-Frankfurt-Munster (AML-BFM) 2004 Registry Protocol and 20 patients with ML-DS 2006 Protocol (Figure 1).

The last patient was enrolled in August 2019, and the last follow-up was done in November 2019. Median observation time was 62.7 months (range, 2.6–174.8 months). Characteristics of the patients are presented in Table 1.

**Table 1:** Characteristics of the patients.

**Characteristic**	**All patients**		**AML-BFM 2004 interim**		**ML-DS 2006**		***p***
	**(*****n*** **=** **54)**		**(*****n*** **=** **34)**		**(*****n*** **=** **20)**		
	***n***	**%**	***n***	**%**	***n***	**%**	
**Sex**							
Male	27	50	19	56	8	60	0.29
Female	27	50	15	44	12	40	
Age at diagnosis	1.9	0.7–17.4	1.9	0.7–17.4	2.2	0.9–4.9	0.26
(median, range), years							
<4	50	92.6	31	91.2	19	95	0.60
>4	4	7.4	3	8.8	1	5	
**History of TAM**							
Yes	15	44.1	8	38.1	7	53.8	0.29
No	19	55.9	13	61.9	6	46.2	
No data	20		13		7		
**FAB**							
M0	5	9.8	5	15.6	0	0	M7 vs. others **0.03**
M1	9	17.6	8	25	1	5.3	
M2	4	7.8	4	12.5	0	0	
M4	1	2	1	3.1	0	0	
M5	1	2	1	3.1	0	0	
M6	2	3.9	1	3.1	1	5.3	
M7	29	56.9	12	37.5	17	89.5	
No data	3		2		1		
**Cytogenetics**							
Trisomy 21 only	21	42.8	14	45.2	7	38.9	Isolated trisomy 21 vs. others 0.65
Trisomy 8	8	16.3	5	16.1	3	16.7	
Del(7)	5	10.2	4	12.9	1	5.5	
Del(16)	3	6.1	3	9.7	0	0	
Del(6)	3	6.1	3	9.7	0	0	
Dup(7)	2	4.1	0	0	2	11.1	
i(7)	2	4.1	1	3.2	1	5.5	
Complex karyotype	6	12.2	4	12.9	2	11.1	
No data	5		3		2		
***GATA1*** **gene mutation**							
Yes	7	38.9	1	20	6	46.2	0.63
No	11	61.1	4	80	7	53.2	
No data	36		30		7		
**Congenital heart defect**							
Yes	24	47.1	16	51.6	8	40	0.25
No	27	52.9	15	48.4	12	60	
No data	3		3		0		
**History before diagnosis, months**							
≤ 3	23	52.3	15	51.7	8	53.3	0.83
>3	21	47.7	14	48.3	7	46.7	
No data	10		5		5		
WBC at diagnosis	6.5	1.9–282	8.83	2.2–282	3.9	1.9–30.9	**0.010**
(median, range), × 10^9^/L							
Platelets at diagnosis	26	2–247	24	2–247	31	9–87	0.52
(median, range), × 10^9^/L							
Peripheral blasts	11	0–92	16	0–92	6.5	0–79	0.24
(median, range), %							
Bone marrow blasts	30	5–91	39	10–91	25	8–59	**0.015**
(median, range), %							

*TAM, transient abnormal myelopoiesis. P-values are bolded where there are significant differences (p < 0.05) between groups*.

The data were collected in PPLLSG AML registry and analyzed retrospectively.

Informed consent to participation in the studies was obtained from guardians of all patients, in accordance with the Declaration of Helsinki. The study was approved by the Ethics Committee of Jagiellonian University Medical College.

From January 2005 to June 2015, 34 patients were treated according to the DS-specific arm of the AML-BFM 2004 Interim Protocol (group I). The treatment consisted of four chemotherapy cycles AIE (cytarabine, idarubicin, etoposide), AI (cytarabine, idarubicin), haM (high-dose cytarabine, mitoxantrone), HAE (high-dose cytarabine, etoposide; additionally intrathecal cytarabine in every cycle, total six doses), and maintenance therapy (6-thioguanine, cytarabine) for 1 year (Figure 1). Compared to the therapy for non-DS AML, there was reduction of doses of antracyclines (idarubicin 8 mg/m^2^ in AIE and 5 mg/m^2^ in AI instead of 12 mg/m^2^ and 7 mg/m^2^, respectively, and mitoxantrone 7 mg/m^2^ in haM instead of 10 mg/m^2^). Median observation time in group I was 91.5 months (range, 38.3–174.8 months).

From June 2015, ML-DS Protocol was introduced. There were additionally three children treated with ML-DS 2006 before 2015 according to individual decision of the treating center. In total, 20 patients were treated according to the ML-DS 2006 Protocol (group II). It consisted of four chemotherapy cycles: AIE (cytarabine, idarubicin, etoposide), AI (cytarabine, idarubicin), haM (high-dose cytarabine, mitoxantrone), and HA (high-dose cytarabine) (Figure 1). Intrathecal treatment with cytarabine was given in every cycle (totally four doses). There was no maintenance treatment. Median observation time in group II was 21.1 months (range, 2.6–89.1 months).

Cytogenetic analyses were performed in local laboratories. Karyotype results were available in 49 patients (91%). From 2015, the status of mutation in *GATA1* gene was done centrally in the Department of Medical Genetics, Institute of Pediatrics, Jagiellonian University Medical College, Krakow, Poland. From each patient, DNA was isolated from 300 μL bone marrow sample collected at diagnosis by the nucleic acid isolation system QuickGene-Mini80 with DNA Blood kit (KURABO Industries Ltd, Osaka, Japan). The variation in *GATA1* gene coding sequence fragments was detected by Sanger sequencing method (3500 Genetic Analyzer; Applied Biosystems, Foster City, California, USA). Separate analyses included 2, 3, and 4 exons of the gene. The *GATA1* gene coding sequence was checked against the reference sequence no. ENST00000376670.7. Results of *GATA1* mutation status was available in 18 patients (33%).

The data that support the findings of the study are available on request from the corresponding author. The data are not publicly available because of privacy or ethical restrictions.

### Statistical Analysis

Descriptive statistical analysis was performed to assess patient baseline characteristics. We used Fisher exact test or a χ^2^ test (categorical variables) and Mann–Whitney *U*-test (continuous variables) for analysis of clinical and laboratory data. Overall survival (OS), event-free survival (EFS), and relapse-free survival (RFS) were calculated using the Kaplan–Meier method. Overall survival was defined as the time from diagnosis to death from any cause; patients alive or lost to follow-up were censored at the date they were last known alive. Event-free survival was defined as the time from diagnosis to disease progression, relapse, or death from any cause. Patients who were alive without disease progression or relapse were censored at the time they were last seen alive and event-free. Relapse-free survival was defined as the time from complete remission (CR) to disease relapse or death from any cause. Patients who were alive without disease relapse were censored at the time of last follow-up. For comparisons of Kaplan–Meier curves, we used the log-rank test. Cumulative incidence of relapse (CIR) was also counted and compared between groups using Gray test. Statistical analysis was performed using STATISTICA 13 software (StatSoft Polska, Krakow, Poland).

## Results

### Patient Characteristics

There were 54 patients enrolled to the study, including 27 girls and 27 boys. The median age at diagnosis was 1.9 years (range, 0.7–17.4 years). Fifty children were younger than 4 years, and four patients were older (4.01, 4.9, 10.5, and 17.4 years). There were no significant differences between groups I and II concerning sex and age. Median number of white blood cells at diagnosis was 6.5 × 10^9^/L (range, 1.9–282 × 10^9^/L). It was significantly higher in group I (median, 8.83; range, 2.2–282 × 10^9^/L) than in group II (median, 3.9; range, 1.9–30.9 × 10^9^/L; *p* = 0.01). Median percentage of peripheral blasts in the whole cohort was 11% (range, 0%−92%), with no significant differences between the groups. The percentage of bone marrow blasts was significantly higher in group I (median, 39%; range, 10–92%) compared to group II (median, 25%; range, 8–59%). Detailed characteristics are presented in Table 1.

### Treatment Outcome

Forty-nine patients (90.7%) achieved CR (30/34 patients in group I and 19/20 patients in group II). Five patients (9.3%) died of toxicities before CR (0.5–2.2 months from diagnosis). The deaths were caused by infections in course of aplasia (two due to sepsis, two due to pneumonia, one due to typhlitis). Forty-five patients (83%) remain in continuous CR (26/34 in period I and 19/20 in period II). There were three deaths in CR (all in group I), two patients died of pneumonia (1 and 7.4 months after diagnosis), and one patient because of cardiac tamponade (1.4 months from diagnosis). In total, eight deaths from toxicities occurred (14.8%): seven in group I (20.6%) and one in group II (5%) (*p* = 0.078). The only one relapse in the whole cohort occurred in the patient from group I, diagnosed of ML-DS at age of 10.5 years. His karyotype revealed isolated chromosome 21 trisomy; *GATA1* mutation was excluded. No additional genetic changes were found in molecular analysis. Leukocyte count at diagnosis was 74.8 × 10^9^/L, with 90% of blasts, much higher than median in the analyzed cohort. The proportion of blast in the bone marrow (91%) was also much higher than median value in the whole group. The patient responded well to the first-line chemotherapy. Relapse occurred 21.7 months from CR. The patient was successfully treated with IdaFLA cycle followed by allogenic hematopoietic stem cell transplantation (HSCT) from the matched sibling donor. Conditioning with fludarabine, melphalan, and total body irradiation was used. The patient received cyclosporine and methotrexate as graft-vs.-host disease (GvHD) prophylaxis. Chronic GvHD with skin involvement occurred in the patient. He remains in the second CR for 33 months.

**Figure 2:**
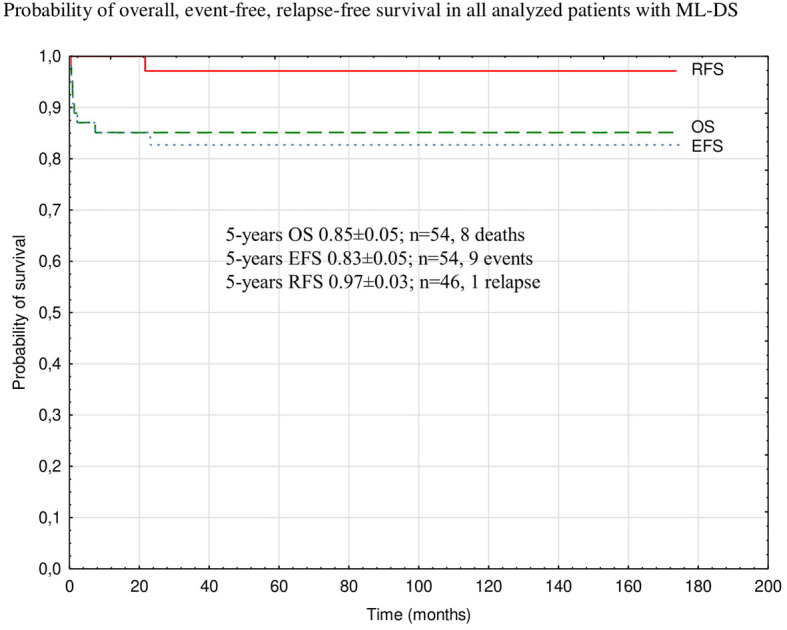
Probability of overall, event-free, relapse-free survival in all analyzed patients with ML-DS. ML-DS, myeloid leukemia of Down syndrome; OS, overall survival; EFS, event-free survival; RFS, relapse-free survival.

**Figure 3:**
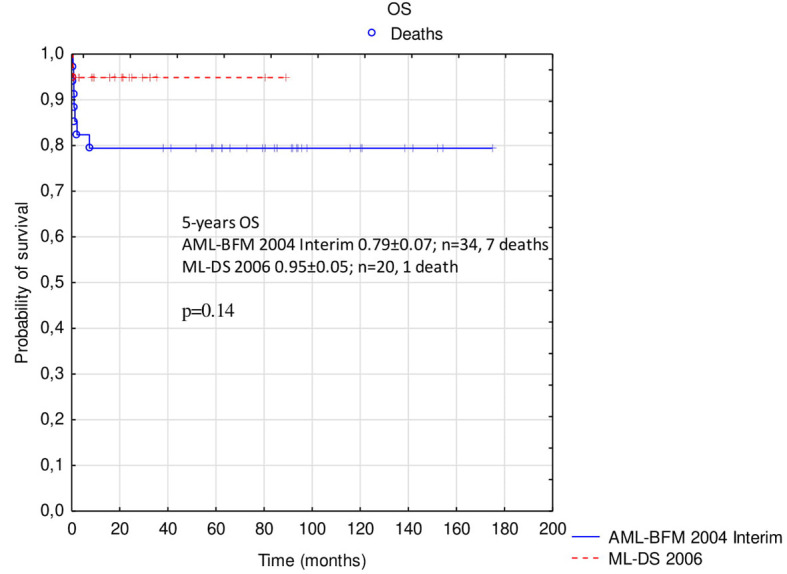
Comparison of overall survival in patients with ML-DS treated with AML-BFM 2004 Interim Protocol and ML-DS 2006 Protocol. ML-DS, myeloid leukemia of Down syndrome; OS, overall survival.

**Figure 4:**
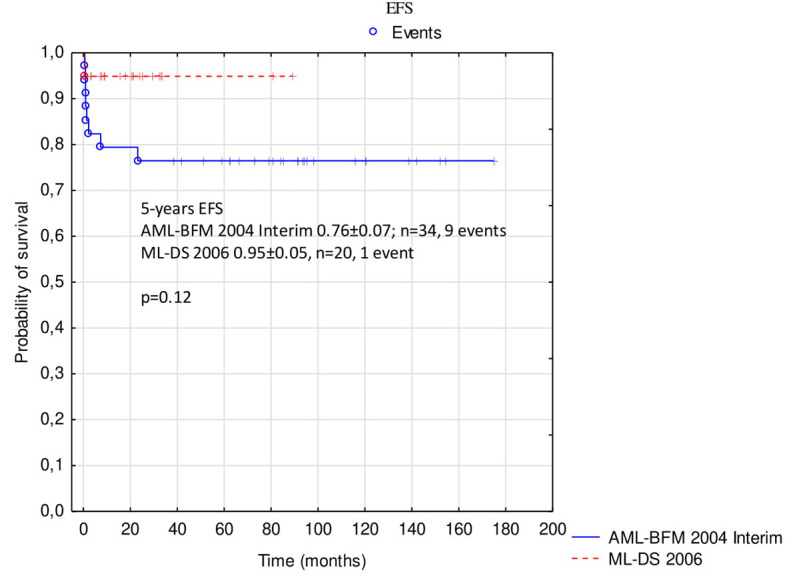
Comparison of event-free survival in patients with ML-DS treated with AML-BFM 2004 Interim Protocol and ML-DS 2006 Protocol. ML-DS, myeloid leukemia of Down syndrome; EFS, event-free survival.

Probabilities of 5-year OS, EFS, and RFS in the whole analyzed group were 0.85 ± 0.05, 0.83 ± 0.05, and 0.97 ± 0.03, respectively (Figure 2). There was a trend toward an improved OS and EFS in group II compared to group I (OS and EFS 0.95 ± 0.05 vs. 0.79 ± 0.07, *p* = 0.14, and 0.95 ± 0.05 vs. 0.76 ± 0.07, *p* = 0.12, respectively; Figures 3, 4). There was one relapse in group I and no relapses in group II. In the whole cohort, 5-year CIR was 0.0286: in group I, 0.037; and in group II, 0. The difference was not statistically significant (*p* = 0.58).

### Genetic Analyses

The *GATA1* gene analysis was available in 18 patients, mainly from group II (*n* = 13). Analysis of *GATA1* gene was not done in the patients who died. Mutation was found in 7 of 18 patients (38.9%). The group of patients with *GATA1* gene mutation did not differ from the group without mutation regarding percentages of blasts at diagnosis and age. There were only two patients older than 4 years with known *GATA1* status, both without mutation. There were no events in the patients with *GATA1* gene mutation and one relapse in the group without *GATA1* gene mutation.

Results of the cytogenetic analysis were available in 49 patients. There were no significant differences in OS nor EFS depending on karyotype (isolated trisomy 21 vs. additional genetic changes). Presence of trisomy 8; chromosome 7, 6, or 16 deletion; or complex karyotype did not influence significant probabilities of OS and EFS (Table 2). Detailed genetic characteristics are presented in Table 3.

**Table 2:** Treatment outcome in the defined groups of the patients.

	**n**	**Deaths**	**OS**	***p***	**Events**	**EFS**	***p***	**Relapse**
Total	54	8	85.5		9	85.5		1
Sex				0.46			0.30	
Male	27	3	0.89		3	0.76		1
Female	27	5	0.81		6	0.89		0
Age at diagnosis, years				0.39			0.72	
<4	50	8	0.84		8	0.84		0
>4	4	0	1.0		1	0.67		1
History of TAM				0.41			0.41	
Yes	15	1	0.93		1	0.93		0
No	19	3	0.84		3	0.84		0
No data	20							
Cytogenetics								
Trisomy 21 only				0.72			0.54	
Yes	21	4	0.84		5	0.80		1
No	28	3	0.87		3	0.87		0
Trisomy 8				0.82			0.74	
Yes	8	1	0.87		1	0.87		0
No	41	6	0.85		7	0.82		1
del(7)				0.66			0.77	
Yes	5	1	0.80		1	0.80		0
No	44	6	0.86		7	0.83		1
del(6)				0.47			0.41	
Yes	3	0	1.0		0	1.0		0
No	46	7	0.85		8	0.82		1
del(16)				0.33			0.42	
Yes	3	1	0.67		1	0.67		0
No	46	6	0.87		7	0.84		1
Complex karyotype				0.29			0.26	
Yes	6	0	1.0		0	1.0		0
No	43	7	0.84		8	0.81		1
No data	5							
Congenital heart defect				0.30			0.51	
Yes	24	2	0.92		3	0.87		1
No	27	5	0.81		5	0.81		0
No data	3							
History before diagnosis, months				0.70			0.45	
≤ 3	23	3	0.87		4	0.81		1
>3	21	2	0.90		2	0.90		0
No data	10							

*EFS, probability of event-free survival; OS, probability of overall survival; TAM, transient abnormal myelopoiesis*.

**Table 3:** Patients with additional genetic abnormalities.

**Cytogenetics**	**Age**,	**FAB**	**Outcome**	**Follow-up, months**
	**years**			
47,XX,+21,del(16)	1–2	7	Death in CR	0.90
47,XY,der(1)add(1)(p?36.3)add(1)(q44),+21c[5] /48,XY,+9,+21c[1]/47,XY,+21c[24]	3–4	0	CCR	85.4
47,XX, +21, add(5),del(1)(q32),del(9)(q13-22),del (12)(q15-24.1)	2–3	7	CCR	32.7
47,XX,dic(5;7)(p12;p12),+21c[4]/47,XX,+21c[10]	1–2	7	CCR	20.7
48-50,XY,+8[10],+8[10],t(18;21)(q10;q10)c, +der(18;21) (q10;q10),+21,+21[2][cp20]	1–2	7	CCR	15.6
48,XY,+8,+21	1–2	1	Early death in aplasia	2.2
47,XY, +21 [3]/48, XY, +8, +21 [2]	2–3	2	CCR	154.4
47,XX,der(5)t(5;15)(q34;q22)+21[7]/47,XX+21[13]	4–5	7	CCR	21.3
48–50,XX,dup(7)(p13p22),+r(21)(pII.2q22.1) × 2–4	2–3	7	CCR	8.1
48,XX, inv(5)(p15q33)del(5)(p15.3),r(7)(p?)del(7) (q22), +21c,+21[10]/47,XX,+21c[15]	2–3	7	CCR	62.7
48,XY,del(6)(q13q22),+11,der(17)t(1;17)(p?;p13), +21c[2]/47,XY,+21c[28]	2–3	7	CCR	38.3
47,XY,r(7)dup(7)(q331q?32),+21c[17]/47,XY+21c[6]	1–2	7	CCR	9.2
50,XY,+19,+20,+21c,+22 [2]/47,XY,+21c[18]	1–2	nd	CCR	66.0
47,XX,+21c,del(6)(q23) (20)	1–2	0	CCR	79.3
48,XX,+8[11],+21[16][cp18]/46,XX[2]	1–2	7	CCR	62.7
47,XY,del(11)(q23),+21c[2]/49,XY,+8,del(11)(q23),+15,+21c[6]/47,XY,+21c[15]	2–3	7	CCR	3.5
47,XX,+21; del(6)(q21qter),del(12)(p12pter),del(5)(q4pter),	4–5	1	CCR	152.1
trisomy 18, trisomy 19, trisomy 22, hyperdiploidy in 25% of metaphases				
47,XX, del(7)(q32),+21c; 47XX, +21	2–3	0	Early death in aplasia	0.6
47,XX, del(16)(q12q22), +21c[11]	<1	7	CCR	120.6
48,XX,+8+21c[5]/47,XX,+21c[12]/46,XX[3]′	2–3	1	CCR	115.8
47,XY,+21c[3]	2–3	7	CCR	18.1
FISH—trisomy 8 in 9% of 200 interphase nuclei				
47,XY,+ 21, i(7q)	1–2	nd	CCR	94.3
47,XX, i(7)(q10), +21c[18]/47,XX, +21c[2]	1–2	nd	CCR	8.8
47,XY,+21c[18]/46, XY [2] FISH del(7q22)(7q35)	2–3	7	CCR	95.4
47,XX, del(16)(q13),t21c [2]/47,XX,+21c	2–3	7	CCR	93.5
47,XY,+21, FISH trisomy 8	1–2	5	CCR	80.5
47,XX,+21,t(7;21)	2–3	7	CCR	89.7
47,XY,+21,der(7)t(7;11)	2–3	7	CCR	80.6

*CCR, continuous complete remission; CR, complete remission; nd, no data*.

### Analysis of Medical History Before Diagnosis

Data concerning medical history from the first symptoms to the diagnosis of ML-DS were available in 34 patients. No significant differences in OS or EFS were found depending on the length of the history (<3 vs. >3 months; Table 2).

In 15 patients (44% from 34 with available data concerning TAM), ML-DS was preceded by TAM. Treatment results were similar in the patients with and without TAM (Table 2).

Congenital heart defect was detected in 24 patients (58.5% of 41 children with available data). In one of those patients with history of atrioventricular canal correction before AML diagnosis, severe cardiac insufficiency occurred during induction therapy. The patient needed second cardiac surgery (closure of secundum atrial septal defect, mitral, and tricuspid valvuloplasty). After stabilization of the general condition, the patient continued chemotherapy and stays in remission. One patient with congenital heart defect died during the treatment because of cardiac tamponade. There were no differences in survival between patients with and without congenital hearts defect (Table 2).

## Discussion

Introduction of the ML-DS–specific treatment protocols with reduced intensity of chemotherapy improved the outcome in that special group of patients (14–17). It resulted in reduction of the risk of toxicities and allowed to maintain excellent treatment response at the same time (14–17). In this study, we analyzed retrospectively the treatment outcome in the two consecutive treatment protocols specific for ML-DS. In AML-BFM 2004 Interim Protocol, the DS-specific arm had reduced antracyclines doses compared to standard treatment, and intrathecal therapy and central nervous system (CNS) irradiation were omitted in the maintenance treatment. In the ML-DS 2006 Protocol, further reduction of chemotherapy was introduced. There was no maintenance treatment; all doses of etoposide were omitted in the last cycle of chemotherapy, and there were four doses of intrathecal cytarabine instead of six in the previous protocol. The treatment outcome in the whole analyzed group of patients (5-year OS 0.85 ± 0.05 and EFS 0.83 ± 0.05) is comparable to the results recently described by large pediatric oncology groups: Children Oncology Group (COG) with 5-year OS 93.0% and EFS 89.9% (15) joined the Nordic Society for Pediatric Hematology and Oncology, Dutch Childhood Oncology Group, and the AML-BFM study group with OS 89 ± 3% and EFS 87 ± 3% (16), as well as Japanese Pediatric Leukemia/Lymphoma Study Group with OS 87.5 ± 3.9% and EFS 83.3 ± 4.4% (17).

In the analyzed group of patients, reduction of the chemotherapy intensity in the ML-DS 2006 Protocol compared to AML-BFM 2004 Interim DS-specific arm resulted in improvement of survival rates (OS 0.95 ± 0.05 vs. 0.79 ± 0.07, *p* = 0.14, and EFS 0.95 ± 0.05 vs. 0.76 ± 0.07, *p* = 0.12), but the differences were not statistically significant. In group I, the treatment-related mortality (TRM) of 20.6% was much higher than described by other authors (1.5–4.9%) (15, 16, 18). Proportion of deaths from toxicities decreased to 5.0% in group II; however, the differences were not statistically significant. Decrease of the TRM probably resulted mainly from improvement of supportive treatment and experience of the treating centers, as almost all deaths from toxicities in group I occurred after the first chemotherapy cycle (reduction of chemotherapy concerned the last chemotherapy cycle and maintenance therapy). Reduction of the intensity of chemotherapy did not affect the treatment efficacy. There were no relapses in group II, compared to one relapse in group I. The 5-year CIR in the whole cohort was 3%. The result is excellent compared to other studies with the 5-year CIR of 6–10.0% (15, 16). Despite generally poor prognosis in relapsed ML-DS (OS, 25.9–34.3%) (15, 19), our patient was treated successfully with IdaFLA chemotherapy followed by HSCT.

In order to optimize the treatment of ML-DS, many efforts are made to find prognostic factors. Uffmann et al. (16) performed multivariate analysis and revealed that poor early response and the gain of chromosome 8 were independent prognostic factors. In the recent study of COG, the only one significant predictor of outcome was MRD on day 28 of induction (15). According to the Japanese Pediatric Leukemia/Lymphoma Study Group AML-D05 study, age at diagnosis of <2 years was a significant favorable prognostic factor for risk of relapse (17). Retrospective international study including 451 ML-DS patients from 13 collaborative study groups participating in the International-BFM AML Study Group revealed that patients with normal karyotype had a higher CIR (21 ± 4%) than cases with an aberrant karyotype (*n* = 255) with a CIR of 9% (±2%) (20).

In our study, no significant prognostic factor was found probably because of low number of patients in the subgroups.

Efforts are made to define mechanisms of leukemic transformation from TAM to ML-DS. In the recent study, Labuhn et al. (21) showed that trisomy 21 and *GATA1* mutation are sufficient for the development of TAM. They identified transforming hotspot mutation in myeloid cytokine receptor *CSF2RB*. Using a multiplex CRISPR/Cas9 screen in an *in vivo* murine TAM model, they found that loss of 18 from 22 tested recurrently mutated ML-DS genes led to leukemia phenotypically, genetically, and transcriptionally similar to ML-DS (21).

The most frequent chromosomal alterations associated with ML-DS are as follows: dup(1q), del(6q), del(7p), dup(7q), +8, +11, del(16q), and +21 (22). In our study among 49 patients with cytogenetic analysis, 43% had isolated trisomy 21. The most frequent numerical abnormality involved trisomy 8 (16.3%); structural abnormalities comprised del(7) (10.2%), del(6) (6.1%), del(16q) (6.1%), dup(7) (4%), isochromosome 7 (4%) (Table 3). Other abnormalities were found in the single patients (Table 3). Complex karyotype was revealed in 12.2% of patients.

The *GATA1* gene analysis became available for most ML-DS patients from 2015. Status of the gene was analyzed in 18 patients. Surprisingly, mutation was found in only 38% of those children, whereas the prevalence of *GATA1* mutation in other studies was 85–89% (15, 16). It could not be explained by low percentage of blasts at diagnosis because it was similar in the group with and without mutation. There could be some false-negative results as the Sanger sequencing used to detect *GATA1* mutation has limited sensitivity. Finally, the group of patients who had *GATA1* analysis was small and could be not representative.

No significant influence of the genetic features on the treatment outcome was revealed in the analyzed cohort.

In conclusion, the study confirms that reduced intensity protocols are very effective in ML-DS patients. The main cause of deaths remained toxicities (there were no deaths from the disease); however, systematic decrease of the TRM was noticed in the analyzed group.

## Data Availability Statement

The datasets generated for this study are available on request to the corresponding author.

## Ethics Statement

The studies involving human participants were reviewed and approved by Ethics Committee of Jagiellonian University. Written informed consent to participate in this study was provided by the participants' legal guardian/next of kin.

## Author Contributions

MCz and WBal designed the study. MCz, KP-W, BS-F, MM, AR-K, AC, JS-S, JW, KM-R, MK-R, DG, JK, LM-K, EA-D, KZ, TU, RT, TS, MN, MW, JP, AC-G, GK, AM-M, KB, WM, KM, WBad, MCi, RC, SS, and WBal were involved in the participates recruitment. TK involved in the laboratory work, and interpretation of its results. MCz, KP-W, BS-F, AR-K, JS-S, KM-R, DG, LM-K, KB, KZ, RT, MN, JP, AC-G, AM-M, MCi, and KM collected the clinical data. MCz involved in the statistical analysis, interpretation of its results, and wrote the first draft of the manuscript. WBal and SS edited the first draft of the manuscript. All authors reviewed the manuscript and approved the final version of the manuscript.

## Conflict of Interest

The authors declare that the research was conducted in the absence of any commercial or financial relationships that could be construed as a potential conflict of interest.
